# Current data processing strategies for cryo-electron tomography and subtomogram averaging

**DOI:** 10.1042/BCJ20200715

**Published:** 2021-05-18

**Authors:** Euan Pyle, Giulia Zanetti

**Affiliations:** Institute of Structural and Molecular Biology, Birkbeck College, Malet St., London WC1E 7HX, U.K.

**Keywords:** cryo-electron microscopy, cryo-electron tomography, subtomogram averaging

## Abstract

Cryo-electron tomography (cryo-ET) can be used to reconstruct three-dimensional (3D) volumes, or tomograms, from a series of tilted two-dimensional images of biological objects in their near-native states *in situ* or *in vitro*. 3D subvolumes, or subtomograms, containing particles of interest can be extracted from tomograms, aligned, and averaged in a process called subtomogram averaging (STA). STA overcomes the low signal to noise ratio within the individual subtomograms to generate structures of the particle(s) of interest. In recent years, cryo-ET with STA has increasingly been capable of reaching subnanometer resolution due to improvements in microscope hardware and data processing strategies. There has also been an increase in the number and quality of software packages available to process cryo-ET data with STA. In this review, we describe and assess the data processing strategies available for cryo-ET data and highlight the recent software developments which have enabled the extraction of high-resolution information from cryo-ET datasets.

## Introduction

Recent developments in cryo-electron microscopy (EM) have enabled the determination of structures of macromolecules at atomic resolution using single-particle analysis (SPA) [1,2]. Obtaining atomic and near-atomic resolution enhances our understanding of the precise mechanisms of protein function and could facilitate structure-based drug design, if sufficient resolution can be reached on clinically relevant drug targets. The ability of EM to image molecules previously intractable by other structural biology techniques, combined with improvements in sample preparation [3,4], data collection [1,5], and processing software [6–8], has dramatically improved our understanding of many classes of proteins such as TRP channels, class-D GPCRs, and bacterial secretion systems [9–12].

Currently, SPA is the favoured cryo-EM method to obtain high-resolution protein structures. However, SPA requires that the protein(s) of interest be purified to a high compositional and conformational homogeneity, that the sample lies in a range of different orientations on the grid, and that individual particles do not overlap with one another. Many proteins do not meet these requirements and thus are not suitable for SPA. Consequently, various studies instead utilise cryo-electron tomography (ET). Cryo-ET utilises a series of tilted two-dimensional (2D) projection images to reconstruct a three-dimensional (3D) representation (or cryo-tomogram) of the sample of interest. This allows the separation of molecules that are stacked or overlapping along the projection view. A cryo-tomogram represents a unique object which can be directly interpreted subject to high level of noise. However, for protein structure determination, individual particles (or subtomograms) can be extracted from the tomogram, aligned to a common reference, and averaged together to generate a structure in a process known as subtomogram averaging (STA). STA improves the low signal to noise ratio (SNR) and has been increasingly used to generate subnanometre resolution structures [13–15]. One distinct advantage of Cryo-ET over SPA is that it can be used to image proteins *in situ* and can, therefore, be used to answer biological questions regarding a protein's native structure and interactions. Cryo-ET and SPA can give insights into structures of proteins embedded in their native cellular environment, also in combination with techniques such as focussed ion beam (FIB) milling for thick samples [16–18]. In addition to obtaining more physiologically relevant structures, the co-ordinates of particles can be annotated into a tomogram so that their interactions with the environment and their geometrical relationship to one another can be visualised.

**Figure 1.**
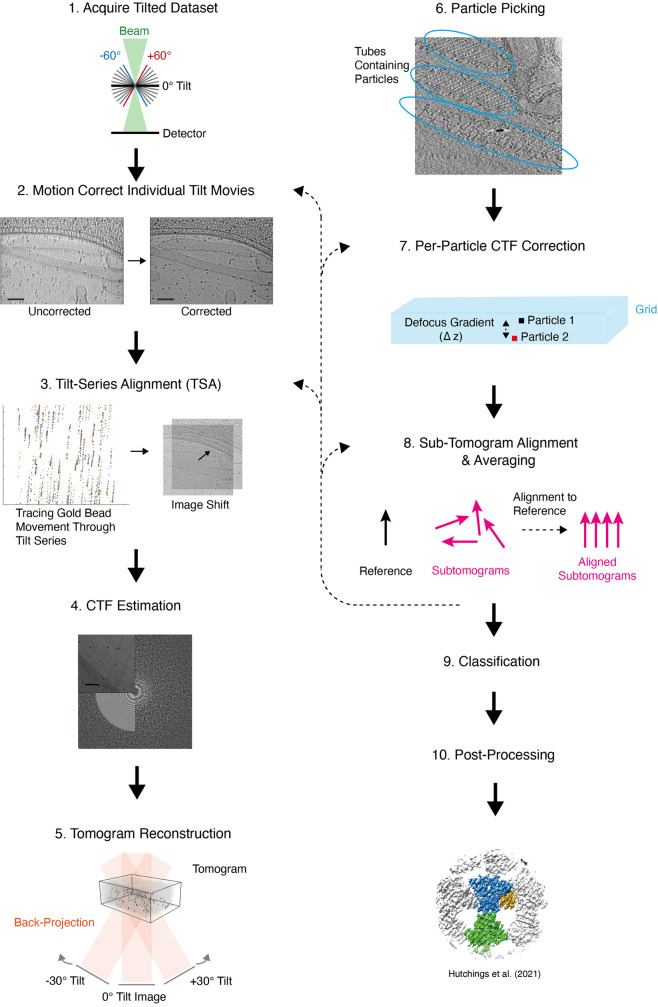
Overview of a typical cryo-ET with STA data processing workflow. Note that several steps can be iteratively refined and/or returned to at a later point in the workflow. The steps that are most commonly iterated are indicated by dotted arrows. The order in which these steps are carried out may vary. (1) During data collection, 2D movies of the sample are captured at fixed points over a range of tilt angles (typically ±60°). (2) Each individual movie is motion-corrected, dose-weighted, and averaged to form a series of 2D images hereafter referred to as a tilt-series. (3) The position of the grid in each image is refined by tracing the position of fiducial markers (usually gold beads) throughout the tilt-series (left) and transforming individual images (right) to ensure a smooth fiducial marker trajectory. This process is known as tilt-series alignment (TSA). (4) Contrast transfer function (CTF) estimation is then carried out for each 2D image in the tilt-series by fitting a CTF model (bottom left) to the Fourier transform (right) of the real image (top left). (5) The 2D images in the tilt-series are backprojected to form a 3D volume, otherwise known as a tomogram. (6) Particles of the protein of interest are picked within the tomogram. Small 3D volumes, or subtomograms, containing one or more particle(s) are then extracted. (7) CTF correction can be carried out at this point or elsewhere in the workflow. (8) The subtomograms are aligned to a reference and averaged. This average is used as a new reference until the alignment reaches convergence and a structure with maximal resolution is generated. The resolution of the resulting structure can be assessed by ‘gold-standard’ Fourier shell correlation (FSC), spectral SNR methods, or local resolution estimations. (9) Conformational and compositional heterogeneity can be assessed by classification of subtomograms after or during STA. This may lead to the generation of multiple structures with different compositions or conformations. (10) Optionally, a range of post-processing techniques can then be used to improve the quality of the structure such as sharpening. The data presented here was generated by Hutchings et al. [19,20].

A brief overview of the steps in a typical cryo-ET and STA data processing pipeline is described in Figure 1; however, it should be noted that this workflow can vary depending on the protein of interest. Additionally, several of these steps are iterative and may be also be repeated and refined at later points in the pipeline. A list of software packages available for each step in this workflow is presented in Table 1 and Table 2.

**Table 1.** Partial list of software used prior to tomogram reconstruction in cryo-ET with STA

	Motion correction	Tilt-series alignment	CTF estimation	Tomogram reconstruction
IMOD [40–42]		✓	✓	✓
Warp [36]	✓		✓	
EMAN2 [44]		✓	✓	✓
Dynamo [46]		✓		
RELION [76]			✓	
emClarity [53]			✓	✓
MotionCorr [30]	✓			
MotionCor2 [32]	✓			
UnBlur [31]	✓			
Alignparts_lmbfgs [33]	✓			
Zorro [34]	✓			
Xmipp [35]	✓			
RAPTOR [43]		✓		
AuTom [45]		✓		
UCSF tomo [47]		✓		
Protomo [48]		✓		
TomoAlign [51] [52]		✓		
CTFFIND4 [70]			✓	
Gctf [71]			✓	

**Table 2.** Partial list of software used after tomogram reconstruction in cryo-ET with STA

	Particle picking	CTF correction	Alignment and averaging	Per-particle refinement	Classification
IMOD [40–42]	✓				
Warp [36]	✓	✓			
EMAN2 [44]	✓	✓	✓	✓	✓
Dynamo [46]	✓		✓		✓
RELION [76]		✓	✓		✓
emClarity [53]	✓	✓	✓	✓	✓
M [54]				✓	
pyTOM [60]	✓		✓		
Chimera (Pick Particle) [62,63]	✓				
DeepFinder [64]	✓				
Xmipp Deep Consensus [65]	✓				
SPHIRE-crYOLO [66]	✓				
NovaCTF [74]		✓			
PEET [83]			✓		
AV3 [85]			✓		
Jsubtomo [86]			✓		

Cryo-ET with STA has increasingly been able to derive subnanometer resolution structures of a range of proteins and complexes such as the COPI, COPII, and clathrin coats [19–22], viruses including HIV and SARS-CoV-2 [23–25], and protein conducting channels [26]. However, cryo-ET with STA does not routinely reach the same resolution as SPA due to several factors. Firstly, the SNR of each image is much lower in cryo-ET than SPA. This is due to the need to image the same particle multiple times during the acquisition of a tilt-series which also requires a reduction the electron dose for each tilt image in comparison with SPA. Additionally, the contrast of the image is reduced at high tilt angles due to increased sample thickness. The low SNR can cause resolution-limiting issues at several points within the STA pipeline such as difficulties in CTF estimation. Secondly, the measured position and the tilt angle of the grid during the acquisition of the tilt-series may be inaccurate due to mechanical instability in the microscope. Tilt-series alignment (TSA) is carried out to detect and correct for inaccuracies in grid position and tilt angle. The TSA must be sufficiently accurate to preserve high-resolution information after tomogram reconstruction. Any inaccuracies will cause the loss of high-resolution information during the back-projection of the individual images to form the tomogram. Finally, it is typically only possible to tilt the grid at a maximum of ±60° before the sample becomes too thick to image effectively due to reduced contrast or to other mechanical impediments, such as the grid support bars. Therefore, during the reconstruction of the tomogram, there are a range of missing views causing resolution anisotropy and the smearing of the tomogram along the direction perpendicular to the tilt axis. This is known as the ‘missing-wedge’ effect. While the anisotropy can be overcome by averaging a sufficient number of particles in each tomogram in a range of different orientations, the missing-wedge can cause inaccuracies in the alignments which ultimately limit the achievable resolution.

Some of the issues limiting the resolution of cryo-ET and STA can be partially solved by advances in data collection protocols. For example, radiation damage over the tilt-series can be distributed in a way which maximises the SNR within the tomogram by using a dose-symmetric tilt scheme [27]. However, some of these problems, such as the missing-wedge effect, cannot currently be resolved by improvements to data collection protocols and are instead being addressed with data processing protocols. This review describes and assesses the various options available for processing cryo-ET data with STA. We also analyse the recent developments in software packages for cryo-ET with STA. We describe how these software packages maximise the quality of the tomograms, and/or structure(s) at each stage along the cryo-ET with STA pipeline. We also discuss the issues that continue to negatively impact the resolution of the structures generated by the cryo-ET with STA workflow.

## Reference-free motion correction

Direct electron detector cameras have enabled the acquisition of images recorded as a stack of dose-fractionated frames or ‘movies’. Over the course of each movie, the images are blurred by the motion of particles within the grid causing the loss of high-resolution information. Several factors influence the change in the positions and orientations of particles during the recording of a movie including mechanical stage drift, Brownian motion, molecular vibration, and beam-induced motion (BIM) such as bending of the support foil and ice [4,28,29]. Mechanical stage drift causes uniform motion of the sample and can be corrected by software which tracks global translational shift between frames of the movie. In contrast, BIM can cause anisotropic, global, and locally correlated movement of particles. For each movie and corresponding 2D image within a tilt-series, much of the particle motion can be detected and corrected for by reference-free motion correction software.

There are several software packages which carry out motion correction of global movements including MotionCorr and UnBlur [30,31]. MotionCorr measures image shifts within the frames of the movie and generates a least-squares estimate of the movements between each movie frame. Whilst this deals well with global translations, it is harder to correct for locally correlated motions of particles induced by BIM. Several software packages, such as MotionCor2, additionally corrects for local non-uniform motions by dividing the movie frames up into tiles and tracking the correlated movements within these subdivisions. Other motion correction software that can account for local motion caused by BIM include alignparts_lmbfgs, Xmipp, and Zorro [32–35].

Major advances in the motion correction implementation have occurred in the years after the introduction of direct electron detectors. More recently, some software packages have integrated incremental improvements in their motion correction algorithms for better accuracy or speed. For example, MotionCor2 v1.4.0 has been developed to improve local motion correction at higher tilt angles [36]. Additionally, Warp is a software package released by Tegunov et al. [37] which carries out all the pre-processing steps in the tomography pipeline, including motion correction. Warp utilises a similar motion correction strategy to MotionCor2 [32,37].

Reference-free motion correction software is often sufficiently accurate at correcting local particle motion in single-particle datasets [32]. However, in tomography, the accuracy and precision of reference-free motion correction is sometimes limited by the low SNR of the images. This issue is more pronounced at higher tilt angles. Furthermore, reference-free motion correction software cannot account for the grid deformations that build up with the acquisition of each subsequent movie within a tilt-series.

## Reference-based motion correction

Reference-based motion correction methodologies have been developed to supplement reference-free motion correction for both SPA and cryo-ET/STA [38–40]. In reference-based motion correction, a 3D reference of the particle of interest is used to generate high SNR 2D projections. In STA, the reference used is typically the subtomogram average generated by the first round of alignment and averaging (see subtomogram averaging). The 2D reference projections are then used to track and correct for the movement of individual particles within low SNR tilt-series frames. As reference-based motion correction conceptually overlaps with the refinement of particle positions on a per-tilt basis it will be discussed below (see per-particle tilt refinement).

## Tilt-series alignment: manual or automated?

TSA is necessary prior to tomogram reconstruction due to inaccuracies in image tracking during data collection. TSA is carried out by detecting and tracking the movement either of high contrast fiducial markers such as gold beads (fiducial-based alignment) or of features in the images such as organelles or membranes (patch tracking or fiducial-less alignment) between tilt images. This data is then used to identify and correct for any inaccuracies in the calculated tilt angle, magnification, or in-plane rotation. Fiducial-based alignment is more accurate and is, therefore, more commonly used when aiming to generate high-resolution structures. The position of the gold beads within each tilt image can be input manually by the user, which represents a time-consuming step sometimes taking multiple weeks for large datasets. However, it is also possible to detect the position of gold beads using automated fiducial marker detection algorithms, which shorten the time taken for this step but may compromise TSA accuracy.

A commonly used tool for TSA is the IMOD software package [41,42]. IMOD allows both manual input of fiducial marker positions and automated marker tracking [43]. Other software can also be integrated with IMOD to carry out TSA using automated marker tracking such as RAPTOR [44]. Several software packages, including Warp and emClarity, integrate IMOD into their workflows for TSA. EMAN2, AuTom, Dynamo, Protomo, and UCSF tomo can be used as alternatives to IMOD for automated TSA using fiducial markers [45–48] or marker-free patch-based alignment [45,46,49].

Many recent improvements have focused on improving the accuracy of automatic detection and tracking of markers so automated TSA can reach the same level of accuracy as time-consuming manual TSA. Automated marker tracking in IMOD was recently improved by implementing several adjustments including the application of a Sobel filter to reduce alignment error, introducing robust fitting to disregard any outliers from the alignment, and by using only the highest-quality markers when an excess of markers is available [43]. Despite these developments, automated TSA sometimes fails [43]. In this case, it is possible to resolve the problem by switching back to manually aligning the tilt images for individual tomograms. Additionally, it is sometimes necessary to remove ‘bad images’ from tilt-series and the appropriate parameters for marker detection may require optimisation thus making the process not fully automatic [43]. AuTom can also perform automated TSA based on the principles presented by Han et al. [50]. AuTom is reported to have a lower failure rate during TSA compared with IMOD and thus require less manual intervention. The EMAN2 software package also includes recent developments in automated patch tracking TSA [45]. EMAN2 uses an iterative approach to TSA which starts with highly binned images. Here, the software selects identifiable ‘landmarks’ from within the tilt-series and refines the TSA. It then generates a binned tomogram with relatively large alignment errors. 2D projections of the 3D landmarks within the tomogram are then used to align the next iteration of TSA, upon which the binning of the tilt images is reduced. As this process is iterated, EMAN2 refines the alignment further and removes outliers. The focus of the TSA in EMAN2 seems to prioritise minimising manual intervention and the increase in data throughput. Chen et al. [45] claim EMAN2 was able to automatically align tomograms where IMOD had previously failed.

Manual input of fiducial marker position remains the most common TSA method for high-resolution STA. Fully automated TSA has not yet become standard practice perhaps due to concerns of resolution-limiting inaccuracies in the detection and tracking of the fiducial markers. However, Chen et al. [45] do not consider the current accuracy of the automated TSA within EMAN2 to be resolution limiting. Additionally, automated TSA has been used in a recent 4.6 Å resolution structure of the COPII inner coat and in a 3.4 Å resolution structure of HIV-1 CA-SP1 hexamer [20,51]. For the COPII inner coat structure, automated TSA routines implemented within the Dynamo software package were coupled with some in-house scripts to minimise the residual error in each tomogram. Furthermore, the accuracy of the initial TSA may be less essential to obtaining high-resolution structures due to recent advances in TSA refinement. Therefore, it is likely we will see increased usage of automated TSA protocols for high-resolution structures in the near future, especially when used in conjunction with TSA refinement tools. Additionally, manual TSA will become a less viable option as data collection for cryo-ET becomes higher throughput and users need to analyse higher numbers of tomograms.

## Tilt-series alignment refinement

Improving the accuracy of the TSA can increase the resolution of maps generated by cryo-ET with STA. TSA refinement can be carried out using either data from later points in the STA workflow or by integrating further steps immediately after the initial TSA. After the initial TSA, TomoAlign builds a model of the deformation of the sample throughout the tilt-series due to BIM. Here, fiducial markers are used to track the deformation of the grid throughout the tilt-series and this information is used to refine the TSA parameters [52,53]*.* Several software packages, such as emClarity and M, provide the user with the opportunity to refine the TSA at the level of individual tilts based on structure(s) generated by STA [54,55]. Due to conceptual overlap we will further discuss TSA refinement in the per-particle tilt refinement section.

## Tomogram reconstruction

After TSA, the individual tilt images are used to produce a 3D reconstruction of the sample. Several methods of tomogram reconstruction can be used. For data being processed by STA, tomogram reconstruction is typically carried out by weighted backprojections (WBP) [56]. Here, each image in a tilt-series is weighted to balance spatial frequencies and then backprojected into a real space volume. Alternatively, the simultaneous iterative reconstruction technique (SIRT) and the similar Simultaneous Algebraic Reconstruction Technique (SART) [57,58] can be used for tomogram reconstruction. In both SIRT and SART, an algorithm is used to optimise the predicted projections of a tomogram and its corresponding tilt images. These methods are aimed at increasing the SNR for direct interpretation of the tomogram compared with WBP. The increased contrast assists processes such as subtomogram alignment and particle picking. However, both techniques lose high frequencies that are determined to have a low SNR. Consequently, high-resolution information is lost in SIRT and SART reconstructions. Therefore, WBP, which preserves high-resolution information, is preferred for generating high-resolution structures by STA.

During tomogram reconstruction, filters can be applied to generate better contrast and lower noise within the tomogram and CTF correction can be performed (see CTF correction). Noisy high tilt images can be low-pass filtered to limit the noise these images contribute to the tomogram. Images can also be filtered according to their accumulated dose over the tilt-series [31].

## Particle picking

In STA, multiple copies of the protein of interest are present within each tomogram. After tomogram reconstruction, it is necessary to locate the positions of the particles within the 3D volume. After the location of the particles has been identified, small 3D subvolumes of each individual particle (or subtomograms) within the tomogram are computationally extracted in preparation for subtomogram alignment and averaging. To generate the highest possible resolution structure from the available data, it is essential to locate as many ‘real’ particles of the protein of interest as possible and to minimise the selection of noise or other molecules as false-positive particles. Alignment of insufficient numbers of ‘real’ particles and excessive numbers of false-positive particles limits the reliability of the structure generated by STA. Cryo-ET data presents additional difficulties in particle picking compared with SPA. For example, *in situ* tomograms are compositionally heterogenous making the protein of interest more difficult to locate. Furthermore, low SNR combined with the missing-wedge effect makes small particles difficult to accurately identify. There are several widely used methods for particle picking in the cryo-ET workflow including manual picking, template matching, geometric surface picking, and machine-learning-based picking.

Several software packages allow manual particle picking including Dynamo, IMOD, and EMAN2 [41,45,47,59]. This involves the user visually inspecting the entire tomogram and selecting positions which they identify as their protein of interest. This requires some *a priori* information on the general shape or location of the protein they wish to investigate. In Dynamo, once multiple particles have been selected, it is possible for the user to manually align the particles which can be useful in generating an initial reference for subtomogram alignment and averaging [47]. However, manual particle picking can be challenging due to noise and to the difficulty of recognising particles in the 3D tomogram on the computer screen. Binning, denoising, and filtering the tomograms can make particles more easily identifiable for manual particle picking. The obvious and major disadvantage of manual particle picking is that it is extremely time-consuming. As a result, manual picking is usually only used to generate an initial reference for template matching and/or subtomogram alignment. However, for some samples other particle picking techniques may be ineffective, leaving manual picking as the only suitable option.

Template matching is an automated method of particle picking which is incorporated into multiple software packages including Dynamo, Warp, pyTom, emClarity, and EMAN2 [37,45,47,54,59–61]. Initially, a template volume must be generated. To manually generate a template volume, a limited number of particles should be manually picked, extracted into subtomograms, aligned, and averaged to produce a low-resolution reference. It is also possible to use a pre-existing structure as a template volume, but care must be taken to avoid reference bias, for example by heavily low-pass filtering the reference [62]. Once a template is available it is used to find particles by cross-correlation. Template matching is a very common method of particle picking as it is a mostly automatic process and it is, therefore, faster than manual particle picking. However, anisotropic resolution due to the missing-wedge effect can limit the identification of particles in some orientations. Furthermore, for complex and compositionally heterogenous tomograms, such as *in situ* data, template matching may produce excessive false positives.

Geometry-based particle picking is a semi-automated method included in Dynamo and the Pick Particle Chimera Plugin [60,63,64]. Here, particle picking is assisted using distinctive geometric features within the tomogram and *a priori* information about the protein of interest. For example, membranes are generally easy to identify within a tomogram and if the protein of interest is known to be membrane associated it is possible to limit particle picking to the surface of the membrane. This has been applied to several biological structures such as virions [23] and coated membrane tubules [19]. Dynamo can be used to annotate a range of geometric shapes including cylinders and spheres within a tomogram [60]. Additionally, surfaces can be manually traced and segmented. The position of the particle relative to the geometric surfaces can also be used to help the initial alignment of the particles. Geometry-based particles picking is particularly useful in complex data such as *in situ* tomograms. It limits the number of false positives by incorporating *a priori* information about particle location. However, similar to template matching, this approach may introduce reference bias if the user is not careful. Moreover, this process is currently only semi-automated as the user has to manually identify the position of the geometric shapes within each tomogram.

Recently, multiple software packages have incorporated machine-learning and deep-learning capabilities into the particle picking process including DeepFinder, Xmipp Deep Consensus, SPHIRE-crYOLO, EMAN2, and Warp [37,65–67]. Most of these software packages have been tailored to SPA datasets; however, some are beginning to incorporate compatibility for cryo-tomograms and *in situ* feature recognition. EMAN2 allows multiple methods for particle picking, including one using neural networks to identify and annotate *in situ* features such as vesicles and microtubules [45,68]. This process does require the user to train the neural network. Here, the user must outline and annotate the feature of interest, such as the endoplasmic reticulum membrane, as well as regions not containing the feature of interest. The neural network can then find and annotate the feature throughout multiple tilt-series and tomograms. Subtomograms can then be extracted from the identified regions. The neural network picking in EMAN2 was able to identify isolated ribosome particles within *in situ* tomograms. These particles could be extracted as subtomograms and were used to generated low-resolution structures via STA [68]. As this process is near-fully automated, it can be carried out with greater speed than the semi-automated geometry-based particle picking. This approach is especially advantageous for *in situ* data where it can take an expert 1 week to fully annotate a tomogram [68]. In contrast, the neural network-based approach takes 2.5 h to annotate a feature on a 2000 × 2000 × 500 voxel tomogram on the workstation used by Chen et al. [68]. Further binning can reduce processing time further. The reported particle picking accuracy of this technique for individual ribosomes varied but was reported to reach as high as 91% [68]. Chen et al. [68] do note some current limitations of the software. For example, gold fiducial markers, carbon edges and other high contrast objects that weren't included in the neural network training data can cause issues with feature identification [68].

DeepFinder is another deep-learning-based particle picking software for cryo-ET datasets which is included in the Scipion package [65]. Similar to EMAN2, DeepFinder requires a neural network to be trained before finding features across multiple tomograms. During benchmarking, DeepFinder outperformed other particle picking approaches [65]. Other software packages are also beginning to incorporate machine-learning particle picking approaches for tomography datasets. For example, the latest release of the machine-learning-based crYOLO (v1.8) has extended support to include cryo-ET data [67]. This feature is currently in the beta phase of testing.

## CTF correction

CTF estimation and correction is carried out in SPA by modelling phase and amplitude oscillations in the Fourier transform of the images. This model can be used to correct the CTF and restore the signal. Accurate CTF correction is essential to recover high-resolution information and generate interpretable structures using cryo-ET with STA [69,70].

The CTF is more challenging to detect and estimate in cryo-ET than in SPA due to the low SNR of individual images, especially at high tilts and high sample thickness, as the Thon rings are less prominent and therefore harder to accurately model. Furthermore, the CTF is modulated by defocus and the tilting of the sample causes a defocus gradient across images. Each image contains an average of all the CTFs across this defocus gradient which dampens the CTF, especially at higher frequencies, which makes accurate detection and estimation more challenging. Inaccurate CTF estimation can lead to the loss of high-resolution information. Several software packages can be used for initial CTF detection and estimation including CTFFIND4, CTFPlotter in IMOD, and gctf [41,71,72]. To improve CTF estimation in cryo-ET, the sensitivity of CTF estimation software must be increased to overcome the low SNR of the individual tilt images.

CTF estimation in cryo-ET is complicated by the defocus gradient across each tilted image. For thin samples, a simple tile-based method is often used to account for the defocus gradient caused by tilting. Here, the defocus can be more accurately estimated by splitting each image in the tilt-series into strips and assigning a different defocus value to each strip [73]. However, for thicker samples, the low SNR in small tiles can cause resolution-limiting errors. Here, the average CTF from each image is estimated and assumed to represent the defocus at the tilt axis.

In both SPA and cryo-ET, particles lie at different heights within the ice. This affects the defocus, and therefore the CTF, of each particle within each image. One advantage of cryo-ET is that it is possible to determine the height, and therefore the defocus, of each particle due to the construction of a 3D volume of the sample. This allows the performance of CTF correction adjusted for particle height, which can be done over the whole tomogram before STA, or at the level of extracted subtomograms during STA.

**Figure 2.**
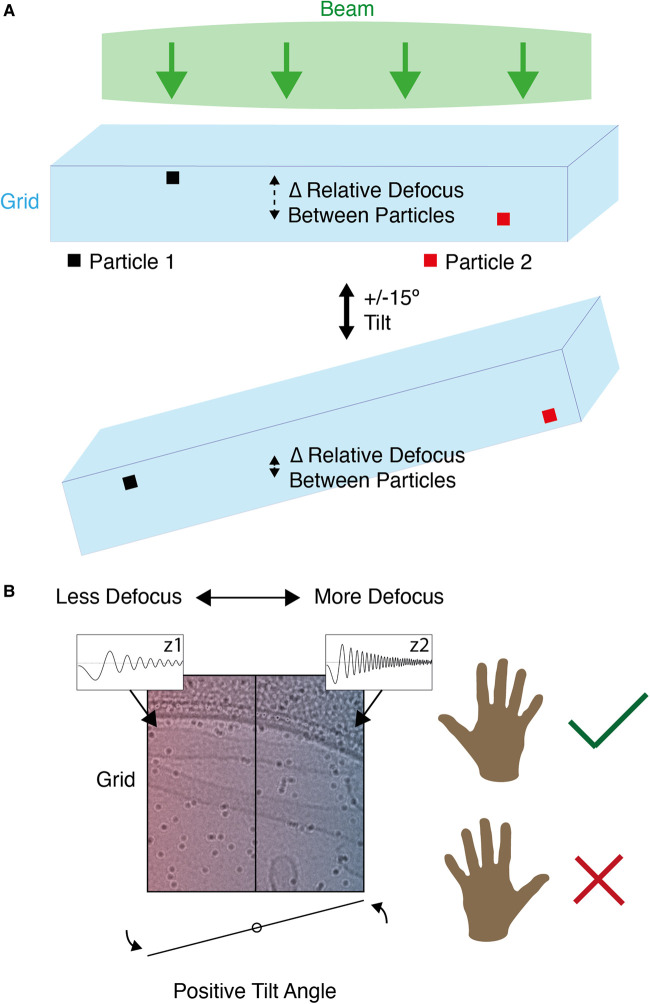
The contrast transfer function in cryo-tomography. (**a**) The defocus of individual particles depends on Z-height in the ice. The Z-height of individual particles is used as part of 3D-CTF correction. Tilting the grid changes the relative defocus of particles at different heights in the ice. (**b**) The defocus gradient across the grid can be used to assess whether the correct handedness is being used. At positive tilt angles, the right side of the grid should be more defocused if the handedness is correct. Z1 shows the defocus of a particle on one side of the grid and its corresponding CTF. Z2 shows the defocus of a particle on the other side of the grid and its corresponding CTF.

Additionally, it is important that the handedness of the system be determined during CTF correction. Most CTF correction software packages make the default assumption that, upon rotating images so that the tilt axis is vertical, their right side is more defocussed for positive tilt angles. If this is not the case, the handedness of the system must be inverted otherwise CTF correction will be inaccurate (Figure 2). This can be done either through a flag in the CTF correction software or by manually rotating tilt images before reconstruction [41,45,74].

NovaCTF is a software package which accounts for sample thickness and different particle heights in CTF correction during tomogram reconstruction [75]. This is commonly known as 3D-CTF correction (Figure 2) [76]. NovaCTF carries out CTF correction for the whole tomogram. 3D-CTF correction can also be done on a per-particle basis where each subtomogram has its CTF corrected individually. RELION carries out per-particle CTF correction where each subtomogram is CTF corrected rather than the whole tomogram [77]. RELION uses the particle co-ordinates to generate a tilt-series of 2D images for each individual particle. The CTF can then be corrected based on the previous defocus calculations, the particle height in the tomogram, and the tilt-series geometry. To avoid interpolation issues, emClarity uses an alternative per-particle 3D-CTF correction approach where the entire tomogram is corrected for the phase of the CTF before tomogram reconstruction but the amplitudes are corrected for each subtomogram after tomogram reconstruction. The CTF correction parameters can be updated after TSA refinement in emClarity (see per-particle tilt refinement) [54].

Warp can also be used for CTF correction of tomography data. Warp models the CTF in image patches which take into account image tilt and local geometry [37]. M can be used in conjunction with Warp to refine CTF parameters [55]. Furthermore, M automatically adjusts the box size of the particles to a sufficiently large size to allow aliasing-free CTF correction which is particularly useful for preserving high-resolution information in particles which have a high defocus. Additionally, M detects and corrects for optical aberrations within the data such as astigmatism, anisotropic pixel size, and higher-order aberrations. The improvement in resolution from correcting these aberrations has previously been established in SPA pipelines [78–80]. The combination of CTF-related features in the Warp/M packages show impressive improvements in obtainable resolution [37,55].

A new software package, CARYON, utilises the comparative information in individual movie frames to obtain more accurate CTF parameters, with the potential to significantly impact our ability to extract high-resolution information from cryo-tomograms [81].

## Subtomogram averaging

**Figure 3.**
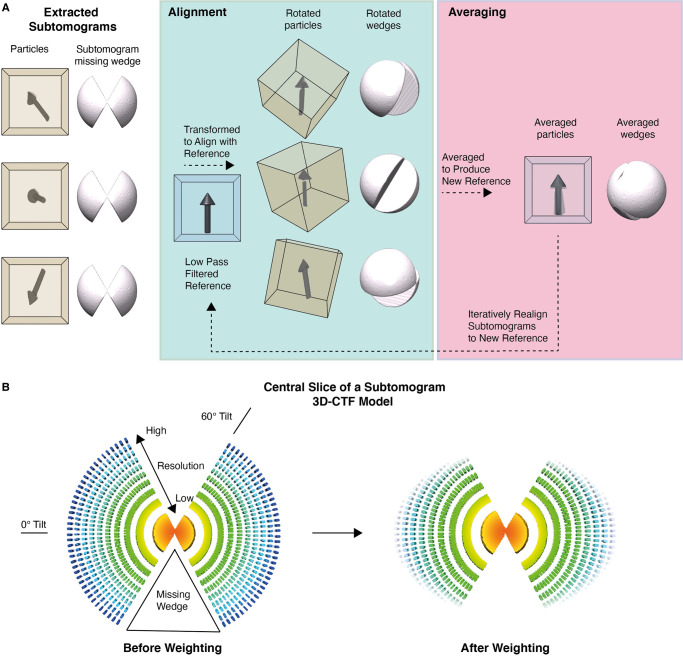
The general process behind subtomogram alignment and averaging. (**a**) The cubes represent randomly orientated extracted subtomograms and the arrows show the orientation of the particles within the subtomograms relative to one another. Each particle has its corresponding missing-wedge representation to the right. (**b**) Visual representation of the 3D-CTF model of an individual subtomogram before (left) and after the high-resolution information loss at high tilts is taken into account by weighting (right). Note the blank areas corresponding to the missing-wedge and the loss of signal at high resolution at higher tilts.

Alignment and averaging of subtomograms produces structures of the complex of interest which have improved SNR compared with the individual particles in the tomogram, and more isotropic resolution (Figure 3). The resolution of the structure generated by STA is affected by the precision and accuracy of the alignment of the subtomograms. The subtomogram alignment and averaging process begins with the alignment of the subtomograms to a common reference (Figure 3a). Similar to template-based methods in particle picking (see particle picking), this reference can be manually derived by hand-picking particles, assigning an approximate orientation, aligning the particles accordingly, and calculating their average. Alternatively, the reference can be generated by using a homologous structure to the protein of interest which is low-pass filtered to avoid overfitting. The reference structure is then rotated in space and aligned to each subtomogram by cross-correlation or maximum-likelihood (ML) methods. The relative orientation of each subtomogram to the reference is stored as metadata, and used to rotate the subtomograms, which are then averaged to generate a new reference (Figure 3a). If alignment was successful, the new reference should have a better SNR than the previous reference which allows more accurate subtomogram alignment. This reference is then used in the next iteration. This alignment and the averaging process is repeated until the reference generated by the averaging step does not significantly improve on the reference used in the previous subtomogram alignment step, otherwise known as convergence. Throughout iterations, parameters such as binning and angular sampling are refined to optimise alignments and processing times.

To avoid overfitting, gold-standard procedures must be used during subtomogram alignment [82]. Prior to initial alignment, the data is split into two halves. The Fourier shell correlation (FSC) between the references generated by each half dataset can be used to estimate the resolution of the references. The estimated resolution of each reference can be used to set an appropriate low-pass filter during alignment, and to assess convergence. Each subtomogram contains a missing-wedge of information which must be taken into account during the alignment process. The effects of the missing-wedge can be partially compensated for by applying a wedge-shaped mask to the reference in Fourier space so the reference has a missing-wedge in a similar position to the subtomogram [83].

A range of software packages can be used to carry out subtomogram alignment and averaging including PEET, EMAN2, RELION, Dynamo, Jsubtomo, AV3/PyTOM, and emClarity [45,47,54,61,84–87]. Recent advances in subtomogram alignment software have focused on improving the missing-wedge compensation in the reference. Most software packages currently use the standard binary wedge-mask method; however, EMAN2, RELION, and emClarity all utilise more advanced methods during missing-wedge compensation which account for the CTF and the SNR in each image over a tilt-series [45,54,85].

In RELION, a 3D model of the CTF of each subtomogram is generated by estimating the 2D CTFs of each image in their tilt-series and then combining this information with the known tilt angles of each image and the Z-height of the subtomogram (Figure 3b). The 3D CTF model describes the missing-wedge in 3D Fourier space (Figure 3b). RELION additionally applies weighting to the 3D CTF model which accounts for the decrease in the SNR of high tilts and the cumulative radiation damage during data collection (Figure 3b). This decreases the contribution of high-resolution Fourier components at high tilts to avoid using noise in subtomogram alignment [85]. Likewise, emClarity iteratively generates a ‘3D sampling function’ for each subtomogram which is similar to the weighted 3D CTF model in RELION and accounts for directional anisotropy. To avoid interpolation between the positive and negative signal at low frequencies, emClarity corrects for the phase of the CTF during tomogram reconstruction and corrects for the amplitude of the CTF during subtomogram alignment.

Warp exports subtomograms alongside 3D CTF volumes indicating the missing-wedge and tilt-dependent weighting information [37]. Warp and M do not carry out STA; however, their workflow has been designed so that RELION can easily be integrated to carry out this step [88]. M allows the user to re-extract and further improve the alignment of particles after its refinement processes. Tools have recently been developed to integrate Warp and M with Dynamo [47,51].

## Per-particle tilt refinement

Once subtomogram alignment and averaging has produced a good-quality map of the protein of interest, it is possible to use this structure to generate high SNR 2D reference projections to refine the position and orientation of particles within the data on a per-tilt basis. This information can be used to refine and optimise several steps previously carried out in the workflow such as motion correction and TSA. The refinement of the parameters within these steps can enable better subtomogram alignment and an improved quality map. Several software packages have recently incorporated per-particle tilt refinement within their STA workflow including M, emClarity, and EMAN2.

The emClarity software package includes a process called tomogram constrained-particle refinement (tomoCPR) which can be used to refine the TSA on a per-tilt basis. Whilst initially emClarity utilises IMOD to generate the TSA parameters and tomograms, tomoCPR is carried out after the initial subtomogram alignment. TomoCPR uses 2D reference projections to identify particles within the individual tilt frames and registers the movements of spatially proximal particles which are artificially clustered together in patches containing a fixed number of particles. Individual clusters of particles within these patches should experience the effects of BIM similarly and emClarity uses this information to smoothen the TSA for groups of neighbouring particles. TomoCPR is also an iterative process which can be refined after each iteration of STA. The improved TSA from tomoCPR allows emClarity to further improve and refine 3D-CTF correction and subtomogram alignment. TomoCPR is demonstrated by Himes et al. [54] as one of the most effective adjustments made within the emClarity workflow. Himes et al. [54] note that tomoCPR is especially effective in tomograms where there are limited numbers of fiducial markers or where the fiducial markers are spatially separated from the particles of interest. It is, therefore, possible that the improvement in TSA is derived from the increased coverage of the grid in fiducial markers which track local changes better, rather than being due to the alignment of previously existing fiducial markers being optimised.

**Figure 4.**
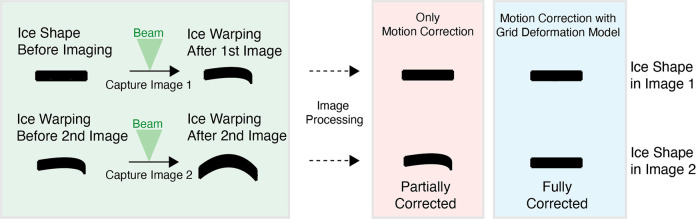
Comparison between correcting for ice deformation during the acquisition of a tilt-series by motion correction or motion correction with a grid deformation model. Note that ice deformation and the accuracy/precision of the software is exaggerated for illustrative purposes.

M is a tool which refines multiple steps within the STA workflow [55]. M is packaged with and utilised alongside Warp. M is used at the end of the STA pipeline to refine several steps across the workflow [55]. It identifies particles in each tilt image through cross-correlation with 2D projections of the reference(s). M accurately tracks the movement of particles and builds a model of the deformation of the grid caused by factors such as ice bending by treating particles as a physically connected group rather than individuals (Figure 4). This allows M to track and correct for the motion of particles caused by the deformation of the grid throughout the entire tilt-series rather than only correcting the motion in individual movies like reference-free motion correction software (Figure 4). Correcting for grid deformation also results in the improvement of the TSA. One of the key advantages of M is that it can identify and track the movement of particles using multiple references. This is particularly beneficial when imaging complex samples such as heterogenous and *in situ* data. Whilst the improvements in obtainable resolution of large ribosome and apoferritin particles demonstrated by Tegunov et al. [55] are promising, future studies will have to demonstrate the applicability of M to smaller particles. Furthermore, the applicability of M to particles which are grouped together in solely in one small region of a tomogram has not yet been established.

EMAN2 also carries out per-particle tilt refinement using the reference generated by the initial STA. Reference projections are used to refine the orientation parameters over a subtilt series generated for each particle and the quality of each tilt is assessed and weighted correspondingly. This corrects for misalignments of particles within the tilt-series, effectively refining the TSA for each particle. Chen et al. [45] report that this subtilt refinement protocol improved the resolution of their ribosome structures from 16 and 13 Å to 8.5 and 9.3 Å, respectively.

It is clear that recent improvements in the TSA refinement can have a major impact on the quality of the reconstructed tomograms. The improvement in tomogram quality that results from an accurate TSA can help refine other steps within the STA workflow, such as subtomogram alignment and 3D-CTF correction. Iterating TSA, 3D-CTF correction, and subtomogram alignment is clearly of strong benefit as refinements of each step in the workflow yield mutual, incremental improvements.

## Classification

Proteins often exhibit both conformational and compositional heterogeneity. Particle heterogeneity can cause the loss of high-resolution information during subtomogram alignment and averaging, particularly within local regions containing structural variability within the data. However, heterogeneity within the data can also be used to elucidate the different states of the protein of interest. Consequently, classification techniques are often applied during or after subtomogram alignment to evaluate the differences between individual subtomograms. This ensures that particles in different conformational or compositional states are not aligned to one another and averaged to a single structure and can be used to separate and derive multiple structures of the same protein in different states. The classification process can also be used to discard subtomograms which either do not contain the particle of interest or are otherwise compromised. Classification can be challenging in cryo-ET due to the effects of the missing-wedge making the differences between individual subtomograms difficult to detect and due to the limited number of particles in some datasets. There are multiple software packages which can classify aligned particles including Dynamo, RELION, emClarity, and EMAN2 [45,47,54,85].

The simplest form of classification is cross-correlation thresholding. This consists of assessing the cross-correlation of each subtomogram to the reference and discarding particles below a specific cross-correlation threshold. This is a fast and easy way to remove ‘bad particles’ from the subtomogram alignment and averaging steps. Care should be taken with the value of the cross-correlation threshold so as not to remove particles which could contribute useful information [19]. Visual inspection of some particles can assist with setting this threshold.

One of the most common methods used to classify particles is principal component analysis (PCA) with k-means or hierarchical clustering [47,54,86]. PCA can be described as a voxel-by-voxel comparison between aligned volumes. K-means or hierarchical clustering are methods of grouping similar images into the same class, effectively separating subtomograms into different groups. Ideally, each group should contain particles of either different conformation, composition, or image quality. PCA is not always suitable for large datasets as it is computationally expensive, in situations where one homogeneous conformation dominates the dataset, or for data where the initial subtomogram alignment fails due to excessive heterogeneity. In multiple reference alignment (MRA) classification, multiple references are generated and the subtomograms are aligned to each reference [47]. The cross-correlation of each subtomogram is calculated for each reference and subtomograms are paired with the reference with which they have the highest cross-correlation. One major shortcoming of MRA is that is requires *a priori* knowledge about the different particle conformations or compositions in the data. ML methods have also been developed to classify subtomograms [89]. The ML statistical framework has been shown to perform better than cross-correlation based methods in situations of poor SNR, avoiding the classification procedure falling into false local minima [90]. ML classification of subtomograms is implemented in RELION [77,85].

EmClarity implements considerable developments in the classification of subtomograms [54]. It utilises its 3D-CTF model (otherwise known as the 3D-sampling-function) to more accurately estimate the effect of the missing-wedge and the SNR on individual subtomograms at higher resolutions. The generation of the 3D-sampling-function was previously discussed in the missing-wedge compensation steps of subtomogram alignment (see subtomogram averaging). emClarity applies the 3D-sampling-function during the classification of each subtomogram which allows higher-resolution information to be utilised in the classification process. This allows the separation of classes of subtomograms with smaller differences than was previously possible. Himes et al. [54] demonstrated the ability of emClarity to distinguish between populations of the 80S ribosomes with subtle conformational differences.

Currently, the classification capabilities of cryo-ET and STA software packages are not as sophisticated as their SPA counterparts. Whilst there have been some recent advances in subtomogram classification, particularly in emClarity, more work is required to improve the classification capabilities of STA software. This is especially critical for *in situ* data in which crowded cellular environments, limited numbers of the particle of interest, noise, and high heterogeneity in the protein(s) of interest can limit the ability to effectively classify subtomograms. This can cause considerable limitations in the achievable resolution.

## Post-processing

After one or more structures have been obtained by subtomogram alignment, averaging, and classification, it is possible to further optimise information derived from the data by using post-processing techniques. For structures generated by STA at a resolution lower than ∼5 Å, it is common for sharpening to be the only post-processing step. During sharpening, the structure factors of the Fourier transformation of the structure are reweighted according to the B-factor of the data [8,88,91].

For higher-resolution STA structures, most post-processing methods are directly adapted from SPA and X-ray crystallography workflows. For example, density modification for cryo-EM has recently adapted from X-ray crystallography by Terwilliger et al. [92,93]. Density modification is used to improve the map according to *a priori* knowledge of the expected structure factors of proteins and solvent. Some STA studies have begun to use density modification to improve the resolution of their maps [20]. Generally, density modification will be most successful where the solvent and particle are clearly separated which may be challenging for *in situ* data. Other post-processing methods which can be used for higher-resolution STA data include LocScale and DeepEMhancer which enhance local sharpening [94,95].

Another recent development in structure refinement has been integrated into the M software package [55]. Prior to reconstructing the final map, M carries out a denoising procedure based on deep learning. Denoising accounts for the local resolution in the generated structure and filters Fourier components to avoid amplifying noise. To estimate the local resolution in the structure, M trains a neural network to recognise and effectively filter noise. Tegunov et al. [55] noted an improvement in resolution isotropy after using their denoising model.

## Discussion

There are several approaches one can take to choose the ideal software and workflow to process cryo-ET data by STA. As different software packages often outperform one another in different areas of the STA workflow, this can introduce challenges in adapting parameters and reformatting the data and metadata to the requirements of different software packages at various points in the pipeline. Recently, more tools have been released to facilitate converting data and metadata to the required format to enable simple interchange between different software packages such as the recent work by Burt et al. [51] which integrates the Warp/M workflow with Dynamo. Reaching the highest possible resolution also often comes at the expense of time as it can require greater computational resources. Consequently, the user should carefully choose their preferred methodology based on a balance of resolution and time. Direct comparison between software packages is not straightforward, as their usage is highly dependent on the type and quantity of data and on the resolution necessary to answer a given biological question. However, it is possible to assess the areas in the STA workflow in which there has been considerable progress and which areas require improvement.

One area in which much progress has been made in recent years is correcting for BIM over a tilt-series, which cause resolution-limiting inaccuracies during TSA and reconstruction. Several software packages have introduced features which correct for this. Specifically, the deformation model in M and tomoCPR in emClarity have both been shown to generate impressive improvements in resolution [54,55]. The continued improvements to automated TSA processes have also reduced the reliance on manual TSA for high-resolution structures which previously represented a time-consuming bottleneck. Use of neural networks in particle picking is another area which shows great potential in both improving the resolution of structures by avoiding picking false-positive particles and by speeding up what was previously another time-consuming bottleneck in the processing workflow. The integration of many steps in the cryo-ET and STA workflow into software packages with GUIs such as EMAN2 and Warp/M has made data processing more accessible to inexperienced users. However, several outstanding issues still remain for processing cryo-tomography data with STA. One of the main issues is data processing speed which can prevents high-throughput data analysis. This will become more critical as advances in data collection, such as fast-incremental single-exposure tilt schemes, increase the speed of acquiring cryo-ET datasets [96]. Whilst several software packages have utilised GPU integration to increase speed [97], processes such as subtomogram alignment can sometimes take considerable computing time and resources. Improving the speed of the STA pipeline will take a combination of increased automation, adapting software to use the most time-efficient methodologies, and improving the ease of use for new users.

There has recently been considerable progress in the field of *in situ* cryo-ET [98]. Analysing the structure of proteins in their native state *in situ* is one of the key goals in structural biology. However, *in situ* data poses specific challenges in areas such as particle picking and classification. Some progress has been made in the area of *in situ* tomogram feature annotation and particle picking by neural networks [68]; however, classification remains an issue particularly for small particles or particles in low abundance. More work will have to be done to extract the full amount of information possible out of *in situ* data. Nevertheless, if the data processing techniques for cryo-ET data with STA consistently improve, it is possible that *in situ* cryo-ET could be used to address previously intractable biological questions.

## Abbreviations

BIM: beam-induced motion
Cryo-ET: cryo-electron tomography
CTF: contrast transfer function
EM: electron microscopy
FIB: focussed ion beam
FSC: Fourier shell correlation
ML: maximum-likelihood
MRA: multiple reference alignment
PCA: principal component analysis
SART: simultaneous algebraic reconstruction technique
SIRT: simultaneous iterative reconstruction technique
SNR: signal-to-noise ratio
SPA: single-particle analysis
STA: subtomogram averaging
TomoCPR: tomogram constrained-particle refinement
TSA: tilt-series alignment
WBP: weighted backprojections
